# Immunotherapy in advanced esophageal squamous cell cancer: earlier or later?

**DOI:** 10.3389/fmed.2025.1524176

**Published:** 2025-07-09

**Authors:** Shuang Wei, Zuoji Li, Tingting Liu, Guizhen Sun, Hongfu Sun, Wei Huang

**Affiliations:** ^1^Department of Radiation Oncology, Gaomi People’s Hospital, Weifang, China; ^2^Department of Science and Education, Gaomi People’s Hospital, Weifang, China; ^3^Department of Nuclear Medicine, Shandong Cancer Hospital and Institute, Shandong First Medical University and Shandong Academy of Medical Sciences, Jinan, China; ^4^Department of Radiation Oncology, Shandong Cancer Hospital and Institute, Shandong First Medical University and Shandong Academy of Medical Sciences, Jinan, China

**Keywords:** immunotherapy, esophageal squamous cell cancer, overall survival, EIT, LiT

## Abstract

**Background and objective:**

Several large-scale phase III clinical trials have confirmed the survival benefit of immunotherapy in patients with locally advanced or metastatic esophageal cancer (EC). The study aimed to investigate whether early use of immunotherapy can improve long-term survival.

**Methods:**

Patients with locally advanced or metastatic esophageal squamous cell cancer (ESCC) diagnosed from January 2018 to December 2021 were retrospectively analyzed. According to the time of immunotherapy, patients were divided into the early immunotherapy group (EIT group, first-line immunotherapy) and the late immunotherapy group (LIT group, second-line immunotherapy). A 1:1 propensity score matching (PSM) was applied to balance the observable potential confounding factors between the two groups. The primary outcome was overall survival (OS).

**Results:**

A total of 359 patients were enrolled; after propensity score matching, the clinical features were well balanced between the two groups, including 107 patients. The median OS was 15.7 months (95%CI: 12.81–18.59) in the EIT group and 17.7 months (95%CI: 14.89–20.57) in the LIT group, respectively (*p* = 0.185, HR = 1.25). The PFS1 of patients was 8.7 months (95%CI: 7.53–9.87) and 7.6 months (95%CI: 5.90–9.30), respectively, and the difference was statistically significant (*p* = 0.032, HR = 0.72). The PFS2 of patients was 12.97 months (95%CI: 11.37–14.58) and 12.93 months (95%CI: 11.65–14.21), respectively, and the difference was statistically significant (*p* = 0.045, HR = 0.73). Subgroup analysis showed that male patients with middle thoracic EC, younger than 65 years old, with only one site of metastasis, only lymph node progression, no combined radiotherapy after progression, and TP (paclitaxel + platinum) regimen chemotherapy may have greater benefits. The COX multivariate analysis showed that the EIT group and the differentiation degree of the tumor had an impact on OS (*P:* 0.03, 0.04; HR: 0.73, 0.70).

**Conclusion:**

Early immunotherapy can improve PFS without affecting OS for patients with locally advanced or metastatic ESCC.

## 1 Background

Esophageal cancer (EC) is one of the most common malignant tumors in the world, and China is a high-incidence area for EC. The morbidity and mortality ranked sixth and fourth among all malignant tumors, respectively. Multiple phase III clinical studies, such as Keynote-181 (1), Attraction-3 (2), Escort (3), and Rationale302 (4), have suggested that, compared with chemotherapy alone, immunotherapy improved overall survival (OS) (from 6.2 months to 10.9 months) and progression-free survival (PFS) (from 1.6 months to 3.4 months) in the second-line treatment of EC. Following, multiple phase III clinical studies, including Keynote-590 (5), Checkmate-648 (6), Escort-1 (7), Orient-15 (8) and Jupoiter-06 (9) have suggested that, compared with chemotherapy alone, the combination of chemotherapy and immunotherapy improved OS (from 9.8 months to 17.2 months) and PFS (from 5.3 months to 7.3 months) in the first-line treatment of EC. Previous studies have shown that the efficacy of immune checkpoint inhibitors is increased in earlier lines of therapy across multiple tumor types compared with in later lines of therapy (10–14). However, it is rarely reported in the real world whether the first-line application of immunotherapy in locally advanced or metastatic EC can bring longer survival benefits. In this study, we retrospectively analyzed the survival of patients with locally advanced or metastatic esophageal squamous cell cancer (ESCC) treated with immunotherapy as the first-or second-line treatment, exploring the value of early application of immunotherapy. This real-world study focused on EC, which has rarely been reported previously, and conducted a subgroup analysis, indicating the population that can benefit from early immunotherapy, which has more guiding significance for clinical medication.

## 2 Materials and methods

### 2.1 Data collection

We retrospectively analyzed patients with locally advanced or metastatic ESCC at Shandong Cancer Hospital from January 2018 to December 2021. The inclusion criteria were as follows: (1) Patients with pathologically confirmed ESCC; (2) Patients initially with unresectable locally advanced or metastatic disease; (3) Patients receiving immunotherapy as first-line or second-line treatment with more than two cycles; (4). Complete imaging data were available for evaluation during treatment or follow-up; (5). Eastern Cooperative Oncology Group (ECOG) score 0–1.

Exclusion criteria: (1) other pathological types of EC, such as adenocarcinoma and small cell carcinoma; (2) Combined with other tumors; (3) Central nervous system metastasis.

According to the time of immunotherapy, patients were divided into the early immunotherapy group (EIT group, first-line immunotherapy) and the late immunotherapy group (LIT group, second-line immunotherapy). The EIT group comprises patients who initially received first-line immunotherapy or progressed to first-line immunotherapy after previous radical treatment. The LIT group was defined as patients who initially received second-line immunotherapy or locally advanced or progressed to second-line immunotherapy after previous treatment. The chemotherapy regimen is paclitaxel or fluorouracil + platinum. The PD-1 inhibitors used among patients included pembrolizumab, toripalimab, sintilimab, envafolimab, and camrelizumab.

### 2.2 Evaluation and follow-up

The primary end point was OS, defined as the time from diagnosis to death from any cause. The secondary endpoints were PFS1, PFS2, disease control rate, and treatment-related adverse events (TRAEs). PFS1 is the time from diagnosis to disease progression or death from any cause. PFS2 is the time from diagnosis to second disease progression or death from any cause. Disease control rate included complete response, partial response, and stable disease. TRAEs were assessed within 90 days after the last dose of medication and were assessed using the Common Terminology Criteria for Adverse Events (CTCAE) version 5.0. The efficacy was evaluated every two courses during the treatment according to the Response Evaluation Criteria in Solid Tumors (RECIST) version 1.1. After the end of treatment, the patients were followed up every three months for 2 years, and once every six months for 3–5 years. Disease progression was assessed by CT scan according to RECIST 1.1 criteria.

### 2.3 Statistical analysis

SPSS 26.0 was used for statistical analysis. Clinical characteristics were compared using the Kruskal-Wallis test for continuous data and the chi-square test or Fisher’s exact test for categorical data. OS and PFS were estimated using the Kaplan–Meier method and compared by the log-rank test. The propensity score-matched analysis (including age, sex, ECOG score, tumor location, differentiation, metastatic sites, number of organs with metastases, chemotherapy regimens, and immune drugs) was performed using the one-to-one nearest neighbor method (ps 0.1). The COX proportional hazards model was used for multivariate analysis to evaluate the possible factors affecting the OS of patients. Statistical results of *p* < 0.05 were considered statistically significant.

## 3 Results

### 3.1 Patients and treatment

**Figure 1:**
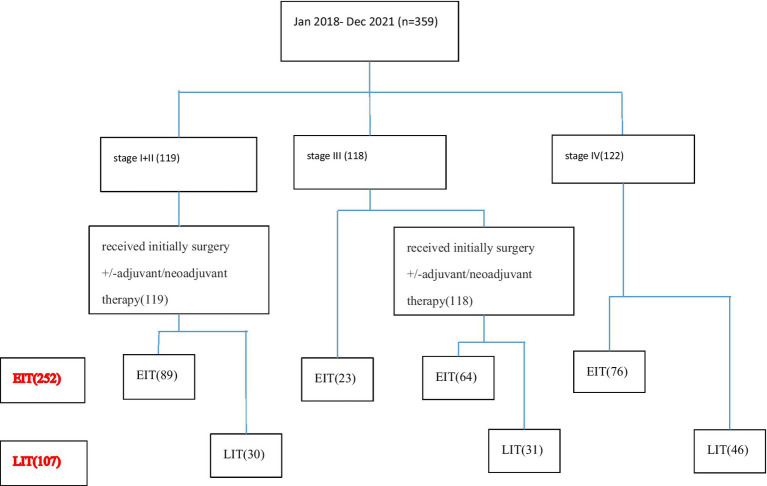
The initial stage and treatment of the patients.

A total of 359 patients with ESCC who met the inclusion criteria from January 2018 to December 2021 were included in the analysis. Among them, 122 patients were at the initial stage IV. Twenty-three patients with initially inoperable locally advanced disease were enrolled in the clinical trial and received first-line immunotherapy and chemotherapy. One hundred nineteen patients were in the early stage (stage I + II) and received radical surgery + adjuvant/neoadjuvant therapy. Ninety-five patients with locally advanced disease who were initially inoperable were treated with radical chemoradiotherapy. According to the number of immunotherapy lines, the patients were divided into two groups: EIT group (252 cases) and LIT group (107 cases). The initial stage and treatment of the patients are shown in Figure 1, the basic characteristics of the patients are shown in Table 1, and the disease control rate is shown in Table 2.

**Table 1:** Basic characteristics of the patients.

Characteristic	EIT group (*n* = 252) (*n*, %)	LIT group (*n* = 107) (*n*, %)	*p* value	EIT group matched (*n* = 107) (*n*, %)	*P* value (psm)
Age, years Median, range	62 (41–82)	61 (42–84)	0.20	62 (42–84)	0.35
Sex			0.95		00.24
Male	222 (88.1)	94 (87.9)		88 (82.2)	
Female	30 (11.9)	13 (12.1)		19 (17.8)	
ECOG performance status			0.90		0.95
0	124 (49.2)	53 (49.5)		53 (49.5)	
1	128 (50.8)	54 (50.5)		54 (50.5)	
Tumor location			0.23		0.17
Cervical segment	10 (4.0)	2 (1.9)		1 (0.9)	
Upper thoracic segment	34 (13.5)	13 (12.1)		11 (10.3)	
Middle thoracic segment	132 (52.4)	48 (44.9)		64 (59.8)	
Lower thoracic segment	76 (30.2)	44 (41.1)		31 (29)	
Differentiated degree			0.92		0.96
High differentiation	14 (5.6)	8 (7.5)		10 (9.3)	
Middle differentiation	73 (29)	30 (28)		29 (27.1)	
Low differentiation	65 (25.8)	27 (25)		28 (26.2)	
Uncertain	100 (39.7)	42 (39.3)		40 (37.4)	
Site of metastasis			0.19		0.54
Liver	39 (15.5)	10 (9.3)		13 (12.1)	
Lung	44 (17.5)	16 (15)		21 (19.6)	
Bone	16 (6.3)	4 (3.7)		6 (5.6)	
Lymph node	153 (60.7)	77 (72)		67 (62.6)	
Number of organs with metastases			0.22		0.26
1	150 (59.5)	71 (66.4)		63 (58.9)	
≥2	102 (40.5)	36 (33.6)		44 (41.1)	
Chemotherapy			0.005		0.66
PF	76 (30.2)	35 (32.7)		33 (30.8)	
TP	151 (59.9)	60 (56.1)		63 (58.9)	
Uncertain	25 (9.9)	12 (11.2)		11 (10.3)	
Immunotherapy			0.001		0.16
Camrelizumab	121 (48)	71 (66.4)		65 (60.7)	
Pembrolizumab	29 (11.5)	2 (1.9)		5 (4.7)	
Sintilimab	63 (25)	24 (22.4)		23 (21.5)	
Envafolimab	7 (2.8)	1 (0.9)		3 (2.8)	
Toripalimab	13 (5.2)	3 (2.8)		5 (4.7)	

Comparison between the LIT group and the EIT group matched for *P* value.

**Table 2:** Short-term efficacy evaluation.

First-line	EIT group (*n* = 252) (immune + chemotherapy)	EIT group (psm, *n* = 107) (immune + chemotherapy)	LIT group (*n* = 107) (chemotherapy)
Complete response	1 (0.4%)	1 (0.9%)	0
Partial response	80 (31.7%)	30 (28.0%)	24 (22.5%)
Stable disease	108 (42.9%)	46 (43%)	53 (50.1%)
Progressive disease	63 (25%)	25 (23.4%)	30 (28%)
Objective response	81 (32.1%)	31 (28.9%)	24 (22.5%)
Disease control	189 (75%)	77 (71.9%)	77 (72.6%)

Second-line	EIT group (*n* = 146) (chemotherapy)	EIT group (psm, *n* = 107) (chemotherapy)	LIT group (*n* = 107) (immunotherapy)
Complete response	0	0	0
Partial response	23 (15.7%)	15 (14.0%)	21 (19.5%)
Stable disease	65 (45%)	50 (46.7%)	39 (36.5%)
Progressive disease	57 (39.3%)	44 (41.1%)	47 (44%)
Objective response rate	23 (15.7%)	15 (14.0%)	21 (19.5%)
Disease control rate	88 (60.7%)	65 (60.7%)	60 (56%)

The baseline clinical characteristics of all patients were comparable after propensity-score matching. The patient’s gender, age, ECOG score, tumor location, degree of differentiation, metastatic site after progression, number of metastatic organs, chemotherapy regimens, different immune drugs used, and whether immunization combined with radiotherapy were analyzed (Table 3). The COX proportional hazard model was used for multivariate analysis to explore the possible factors influencing OS.

**Table 3:** Multivariate analysis.

Variables	*p*-value	HR (95%CI)
Sex	0.69	0.91 (0.56–1.46)
Age	0.11	0.78 (0.58–1.05)
ECOG	0.72	0.95 (0.73–1.24)
Location	0.87	0.98 (0.81–1.19)
Differentiation	0.04	0.70 (0.50–0.99)
Site of metastases	0.82	1.01 (0.92–1.11)
Number of organs with metastases	0.07	1.25 (0.98–1.60)
Chemotherapy regimens	0.22	1.11 (0.94–1.31)
Immunotherapy regimens	0.44	1.04 (0.94–1.15)
Immune with or without radiotherapy	0.19	1.20 (0.91–1.58)
EIT group or LIT group	0.03	0.73 (0.55–0.98)

### 3.2 Survival

All patients were followed regularly until November 30, 2022, or death from any cause. The median duration of follow-up was 26.8 months in EIT group and 29.9 months in LIT group. In EIT group, 90 (35.7%) patients did not progress after first-line immunotherapy + chemotherapy; seven patients (4.8%) did not progress after second-line chemotherapy. After the progression of first-line immunotherapy + chemotherapy, 16 patients (6.3%) did not receive second-line chemotherapy. Among them, five patients (2.0%) did not receive second-line treatment due to death, and 11 patients (4.4%) were unable to accept or refused second-line treatment due to poor health. In LIT group, all patients received second-line immunotherapy after progression on first-line therapy, and 16 patients (15%) were still receiving second-line immunotherapy without tumor progression as of the follow-up date. Of the patients who subsequently entered third-line therapy, 15.5% were in the EIT group and 30% were in the LIT group.

**Figure 2:**
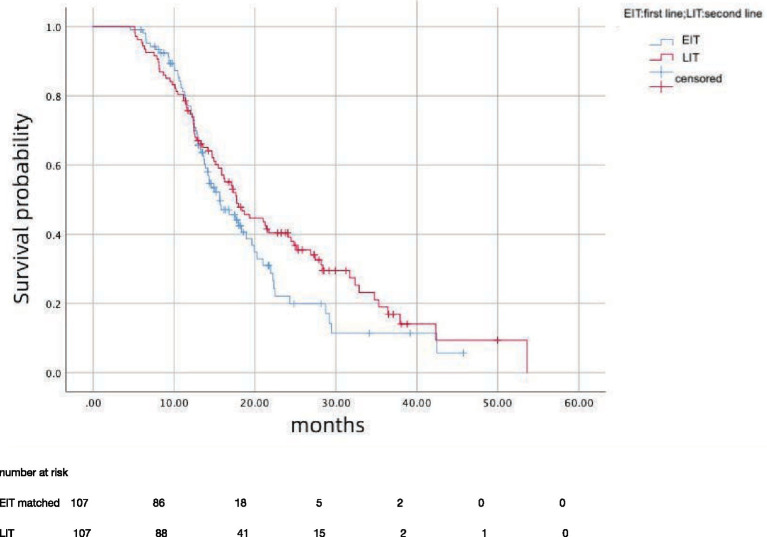
Overall survival (months).

**Figure 3:**
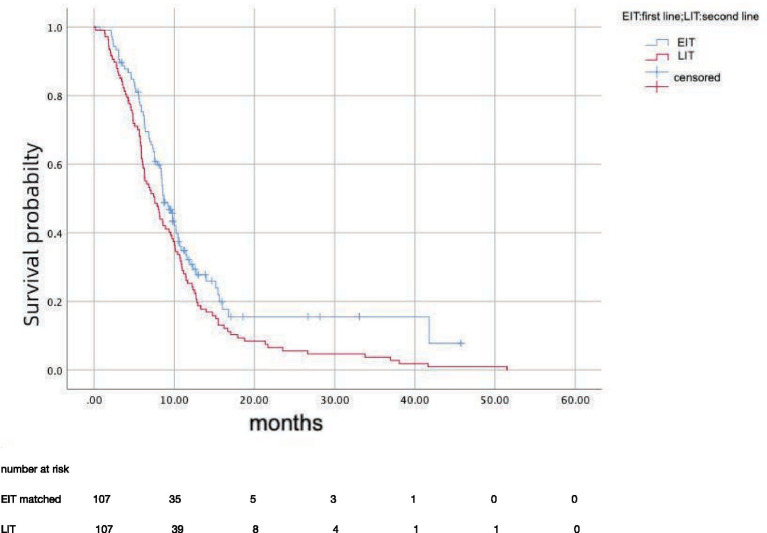
Progression-free survival 1(months).

**Figure 4:**
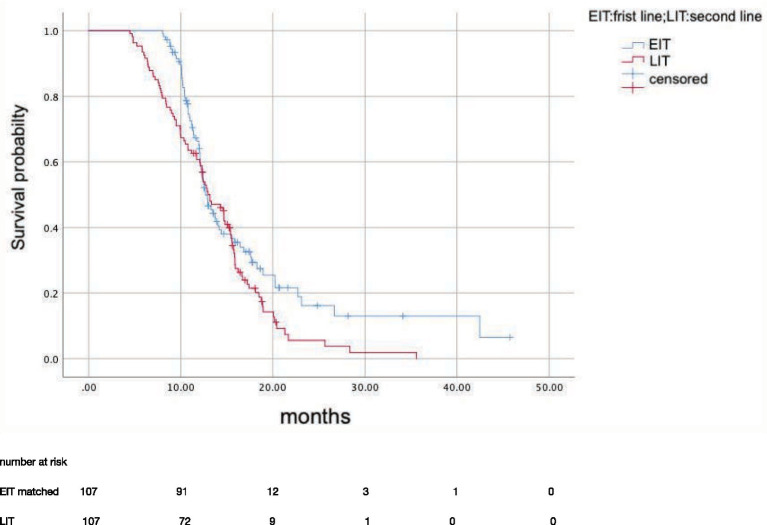
Progression-free survival 2(months).

After propensity score matching, 107 patients in EIT group were matched to those LIT group; the median OS was 15.7 months (95%CI: 12.81–18.59) in EIT group and 17.7 months (95%CI: 14.89–20.57) in EIT group, respectively, with no statistically significant difference (*p* = 0.185, HR = 1.25) (Figure 2). The median PFS1 of the two groups was 8.7 months (95%CI: 7.53–9.87) in the EIT group and 7.6 months (95%CI: 5.90–9.30) in the LIT group, with a statistically significant difference (*p* = 0.032, HR = 0.72) (Figure 3). The median PFS2 of the two groups was 12.97 months (95%CI: 11.37–14.58) in the EIT group and 12.93 months (95%CI: 11.65–14.21) in the LIT group, with a statistically significant difference (*p* = 0.045, HR = 0.73) (Figure 4).

**Figure 5:**
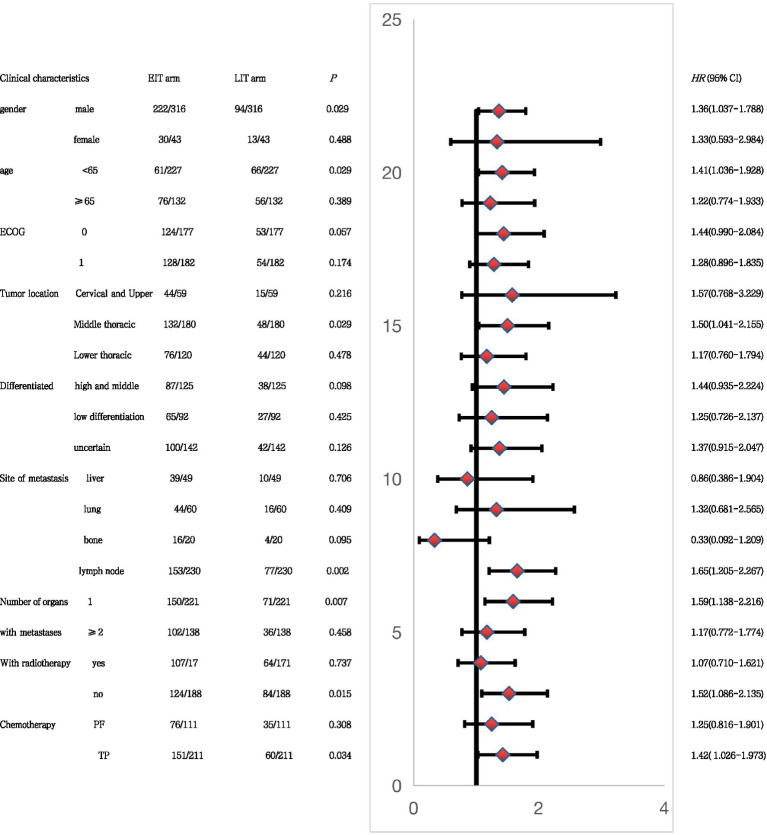
Subgroup analysis.

Subgroup analysis using PFS2 as the end point (Figure 5) showed that in male patients, younger than 65 years of age, with esophageal tumors located in the middle thoracic segment, lymph node metastasis after progression, and one organ metastasis. First-line treatment without combination radiotherapy, and a TP regimen (paclitaxel and platinum) combined with chemotherapy, and the combination of immunotherapy in the first-line treatment may have greater benefits than the second-line combination of immunotherapy. This also provides a reference for our clinical treatment options.

The COX proportional hazard model was established, and multivariate analysis showed (Table 3) that the EIT group (*p* = 0.03, HR = 0.73) and differentiation degree of the tumor affected OS (*p* = 0.04, HR = 0.70). However, gender, age, ECOG score, tumor location, metastatic site, number of metastatic organs, chemotherapy regimen, immune drugs, and whether immunotherapy combined with radiotherapy had no significant effect on OS. Treatment-related adverse effects are shown in Table 4. In this retrospective data, 122 patients with stage IV were selected and divided into two groups, in addition to radiotherapy as part of the initial treatment: 67 patients with radiotherapy and 55 patients without radiotherapy. The median OS was 17.8 months and 15.8 months, respectively (*p* = 0.179).

**Table 4:** Adverse events related to treatment.

Adverse events	No. (%) of patients					
	EIT group (*n* = 252)		EIT group (psm, *n* = 107)		LIT group (*n* = 107)	
	Any grade	≥ Grade 3	Any grade	≥ Grade 3	Any grade	≥ Grade 3
Treatment-related adverse events	249 (98.9)	152 (60.4)	104 (97.2)	64 (59.8)	104 (97.0)	67 (62.5)
Anemia	193 (75)	42 (16.5)	78 (72.9)	16 (15)	79 (74.2)	13 (12.5)
White blood cell counts decreased	157 (77.8)	63 (25)	82 (76.6)	25 (23.4)	75 (70.3)	27 (25.6)
Neutrophil count decreased	167 (66.5)	79 (31.2)	73 (68.2)	35 (32.7)	80 (63.5)	48 (45.4)
Nausea	132 (52.4)	4 (1.5)	54 (50.4)	2 (1.9)	55 (51.3)	2 (1.7)
Asthenia	122 (48.5)	6 (2.2)	48 (44.9)	2 (1.9)	46 (43.4)	3 (2.5)
Decreased appetite	107 (42.5)	1 (0.5)	46 (43)	1 (0.9)	47 (44.1)	2 (1.5)
Vomiting	89 (35.5)	6 (2.4)	33 (30.8)	2 (1.9)	34 (31.7)	2 (2.0)
Platelet count decreased	64 (25.5)	5 (2.0)	25 (23.4)	2 (1.9)	25 (23.6)	2 (2.0)
Weight decreased	60 (23.8)	2 (0.7)	24 (22.4)	3 (2.8)	23 (21.6)	2 (2.0)
Aspartate aminotransferase increased	30 (12.1)	3 (1.0)	10 (9.3)	1 (0.9)	11 (10)	1 (1.0)
Immune-related adverse events	252 (84.6)		34 (31.8)		98 (33.0)	
Reactive capillary endothelial proliferation	121 (48)		48 (44.9)		71 (66.4)	
Hypothyroidism	31 (12.3)		9 (8.4)		7 (6.5)	
Hyperthyroidism	6 (2.4)		2 (1.9)		1 (1.0)	
Rash	16 (6.4)		6 (5.6)		2 (2.0)	
Pneumonitis	16 (6.5)		5 (4.7)		2 (2.0)	

## 4 Discussion

Treatment outcomes are poor for patients with advanced disease. The median OS rates are ~10 months and ~6 months for first-line and second-line chemotherapy, respectively, with objective response rates of ~30% and ~10%, respectively (15). With the advent of the immune era, immunotherapy has gradually become the standard treatment for advanced EC. Keynote-181 (1) confirmed the efficacy of pembrolizumab in PD-L1 cps ≥ 10 in patients with locally advanced EC; there was a two-fold improvement in survival at 12 months (43% vs. 20%) compared with chemotherapy alone (fluorouracil combined with platinum). Therefore, in 2019, it became the first immune drug approved for second-line treatment of advanced EC in the United States. Attraction-3 (2) confirmed that at a minimum follow-up time (i.e., time from random assignment of the last patient to data cutoff) of 17.6 months, OS was significantly improved in the nivolumab group compared with the chemotherapy group (10.9 months vs. 8.4 months, HR = 0.77, *p* = 0.019).

With the overall success of immune drugs in second-line therapy, their use is gradually advancing to first-line therapy. Keynote590 (5) reported that first-line palivizumab + chemotherapy (fluorouracil + platinum-based) vs. first-line chemotherapy in locally advanced or metastatic EC, regardless of the expression status of PD-L1 CPS, showed that the first-line immunized group improved OS (12.4 months vs. 9.8 months; 0.73 [0.62–0.86]; *p* < 0.0001) and progression-free survival (6.3 months vs. 5.8 months; 0.65 [0.55–0.76]; *p* < 0.0001), demonstrating a significant benefit. The escort-1 (7) trial confirmed that first-line immunization + chemotherapy (paclitaxel + cisplatin) was better than chemotherapy alone (paclitaxel + cisplatin) in terms of both OS and progression-free survival in the Chinese population, which were 15.3 vs. 12.0 months, respectively (HR = 0.7) and 6.9 vs. 5.6 months (HR = 0.56). The Orient-15 (8) trial also demonstrated a survival benefit for Sintilimab in first-line chemotherapy (paclitaxel + cisplatin/fluorouracil + cisplatin). Overall survival (median 16.7 vs. 12.5 months, HR = 0.63, 95%CI 0.51–0.78, *p* < 0.001) and progression-free survival (7.2 vs. 5.7 months, HR = 0.56, 95%CI 0.46–0.68, *p* < 0.001).

Our retrospective data show that after PSM, the OS of 15.7 months with first-line immunotherapy + chemotherapy in locally advanced or metastatic ESCC is consistent with the OS reported with camrelizumab (15.3 months) and sintilizumab (16.7 months), which is higher than the OS reported with pembrolizumab. One possible explanation for this discrepancy may be that a smaller proportion of patients in the control arm of the Keynote-590 study had been exposed to second-line immunotherapy. In terms of PFS, our data showed that the median PFS1 of the two groups was 8.7 months and 7.6 months, respectively (*p* = 0.032, HR = 0.72), which was similar to the HR values of the above three reports (0.65, 0.56, 0.56). The median PFS2 of the two groups was 12.97 months in the EIT group and 12.93 months in the LIT group, with a statistically significant difference (*p* = 0.045, HR = 0.73).

Previous reports showed that in non-small cell lung cancer, patients previously treated with fewer lines of therapy (i.e., in the first-line setting) might have less refractory and immunosuppressive tumor microenvironments than patients who have progressed on therapy (16). Pembrolizumab + chemotherapy in the Keynote-189 study reduced the risk of death by 44% in metastatic non-squamous NSCLC (17). Nivolumab as a second-line treatment reduced the risk of death by 27% in metastatic non-squamous NSCLC (18). The keynote-059 (14) trial showed that pembrolizumab monotherapy was effective, safe, and well tolerated in locally advanced gastric or gastroesophageal junction cancer with at least two previous lines of therapy, regardless of PD-L1 expression.

So, whether the early application of immunotherapy is more effective is still controversial. It is also rarely reported whether first-line immunotherapy has a greater survival benefit than second-line immunotherapy in treating locally advanced or metastatic EC. Our retrospective real-world study showed no significant difference in OS between the use of first-line immunotherapy + chemotherapy and chemotherapy alone in patients with locally advanced or metastatic EC, but a benefit in progression-free survival was observed with the addition of immunotherapy to the first-line regimen.

Our study showed that there was no significant difference in OS between the two groups, which we believe is mainly because twice as many patients in the LIT group compared to the EIT group received third-line therapy by the date of follow-up (15.5% vs. 30%), which resulted in significantly longer OS in the LIT group. However, there were statistically significant differences in PFS1 and PFS2 between the two groups, further indicating the benefit of immune drugs in treatment; the benefit was more significant in the early application.

In EIT groups, our subgroup analysis showed that male patients with middle thoracic EC, younger than 65 years old, with only one site of metastasis, only lymph node progression, no combined radiotherapy after progression, and TP (paclitaxel + platinum) regimen chemotherapy had better progression-free survival. In clinical practice, for young patients with lymph node metastasis or single organ metastasis, or when local radiotherapy cannot be added in time, immunotherapy should be given to patients in a timely manner.

Fluorouracil combined with cisplatin is commonly used in combination chemotherapy in Western countries, while paclitaxel combined with platinum is preferred in China (19, 20). Our retrospective data also showed that more patients chose the paclitaxel + platinum regimen. Previous retrospective reports (21) showed no significant difference in the efficacy of the two regimens in EC. However, our subgroup analysis suggests that the TP (paclitaxel + platinum) regimen is preferred as the chemotherapy regimen when combined with immunotherapy.

Li et al. (22) reported for the first time the difference in survival between first-line immunotherapy + chemotherapy and chemotherapy alone in locally recurrent or advanced metastatic esophageal squamous cell carcinoma. In their retrospective study, there was no significant difference in OS (13.5 vs. 13.1 months, *p* = 0.7) between immunotherapy + chemotherapy and chemotherapy alone, while PFS1 was significantly different (7.1 vs. 4.1 months, *p* = 0.001, HR = 0.53). Our retrospective data showed that the OS of first-line immunotherapy combined with chemotherapy and chemotherapy alone was 15.7 months and 17.7 months, respectively (*p* = 0.185, HR = 1.25). PFS1 was 8.7 months and 7.6 months, respectively (*p* = 0.032, HR = 0.72), consistent with the above conclusions. However, compared with this retrospective study, the number of patients in our article is larger, the follow-up time is longer, and the previous treatment history of the enrolled patients is more detailed, complex, and closer to the actual clinical treatment. Furthermore, we conducted subgroup analyses to inform our practice of which patients would be more inclined to be treated with immunotherapy in the first-line treatment.

### 4.1 Advantages and defects

#### 4.1.1 Advantages

In this retrospective study, we found that the combination of immune therapy and chemotherapy is more advantageous than chemotherapy alone in the first-line treatment of patients with locally advanced or metastatic EC. Furthermore, our real-world study included a larger number of cases and was closer to the actual clinical treatment situation than the study by Li et al. (22). The results of subgroup analysis also hold certain guiding value for our clinical practice.

#### 4.1.2 Defects

This article is a single-center, retrospective study, and the data may have a certain loss bias. The short follow-up time and some patients still in treatment may have a certain impact on the calculation of survival time. In this retrospective study, PD-L1 expression status was unknown in most patients, and the effect of PD-L1 expression level on survival could not be assessed. Our study included only esophageal squamous-cell carcinoma and not adenocarcinoma, which has a very low incidence, and therefore has no significant value in guiding the clinical management of esophageal adenocarcinoma.

## 5 Conclusion

For patients with locally advanced and metastatic EC, early application of immunotherapy has a progression-free survival benefit. In clinical practice, patients with middle thoracic EC, younger than 65 years old, with only one site of metastasis, only lymph node progression, no combined radiotherapy after progression, and TP (paclitaxel + platinum) regimen chemotherapy are inclined to be treated with immunotherapy in the first-line treatment.

## Data Availability

The raw data supporting the conclusions of this article will be made available by the authors, without undue reservation.
